# New Approaches to Assess Fertility in Domestic Animals: Relationship between Arterial Blood Flow to the Testicles and Seminal Quality

**DOI:** 10.3390/ani11010012

**Published:** 2020-12-23

**Authors:** Ana Velasco, Salvador Ruiz

**Affiliations:** 1 Department of Physiology, Faculty of Veterinary Medicine, Campus Mare Nostrum, University of Murcia, 30100 Murcia, Spain; ana.velascor@um.es; 2 Institute for Biomedical Research of Murcia, IMIB-Arrixaca, 30120 Murcia, Spain

**Keywords:** breeding soundness evaluation, ultrasonography, doppler mode, testicular artery, fertility, resistive index, pulsatility index

## Abstract

**Simple Summary:**

Reproduction is one of the most important economic factors at a production level. For that reason, the evaluation of the breeding soundness (BS) is of great relevance to secure high production rates. There is ongoing research to find new markers to bring closer breeding soundness examination (BSE) results to real fertility outcomes. Among the different methods to evaluate BS, ultrasonography is routinely used and it is the method of choice to evaluate the testicular parenchyma. Doppler mode allows the quantitative and qualitative evaluation of the irrigation of organs and can be used as an important diagnostic technique for evaluating the testicular irrigation. In humans and several animal species, a relationship between Doppler parameters of the testicular artery and seminal quality has been found. This suggests pulsed wave Doppler ultrasound measurements and indices could be proposed as objective parameters to evaluate testicular function.

**Abstract:**

The early identification of infertile males improves reproduction efficiency at a production level and is essential to secure high production rates. Before entering a breeding program, males must pass an initial breeding soundness examination (BSE) which consist of several diagnostic exams whose end point is to estimate their future fertility. There is ongoing research to find new markers that allow better identification of fertile males. Doppler mode allows the quantitative and qualitative evaluation of the irrigation of organs. When evaluating the reproductive system, Doppler mode has been successfully used for the evaluation of the uterine and ovarian irrigation. In males, it is gaining relevance for the evaluation of testicular irrigation. Researchers have found a relationship between pulsed-wave Doppler velocimetric parameters and seminal quality in various domestic animal species. This suggests Doppler ultrasound parameters should be considered as objective parameters to evaluate testicular function. In this review, we analyze the results in the main domestic animal species and discuss the differences and similarities among the results. We also discuss the effect of the location of the measurements, breed, season and laterality in the measurement of Doppler velocimetric parameters as well as the impact and limitations of this method of assessing breeding soundness.

## 1. Introduction

Reproductive technologies dictate the strategies that can be used to select genetically for traits that improve production. Of critical importance has been artificial insemination (AI). Given the central role of reproduction as a determinant of production efficiency and genetic selection in breeding programs, improvements in reproductive technologies will be crucial to face the challenges created by the increase in world population [1]. Reproduction is one of the most important economic factors in the livestock industry, based not only on the reproductive capacity of the female, but the male as well [2]. Farming systems’ success depends on livestock management, especially improved reproductive performances. Both female and male genitals systems have to be sound to ensure successful breeding [3]. Male Breeding Soundness Evaluation (BSE) is an important component and common practice in domestic farming species. It is meant to predict the breeding capacity of males and enables us to identify and withdraw infertile males from the breeding program, selecting the best performing individuals as sires [4,5].

Contemporary guidelines for BSE mainly center upon such aspects as breeding history, general health, physical examination, scrotal circumference and sperm morphology and motility [5,6,7,8]. Thus, the evaluation of semen quality is one of the major evaluations for the selection of males for breeding. Methods to assess the quality of semen before the male starts its reproductive life are required to predict their fertility potential [7]. Physical examination such as palpation of the testis has been proved inefficient when accurately assessing the testicular parenchyma and the presence of small lesions [5]. Increasingly, B-Mode ultrasonographic evaluation of reproductive organs is routinely used, as it is a safe, non-invasive technique for the identification of a normal testicular parenchyma, and it can be an important tool for the evaluation of testicular biometric parameters, which have been correlated with fertility potential [3,7,8].

Due to the importance of BSE for the election of the best performing males and its direct effect in the flock fertility, it is necessary to develop other fertility markers for early BSE to improve productivity and genetic gain [4]. Testicular hemodynamic is the main route for the transport of oxygen, nutrients and other hormones to and from the testis [9]. Testicles receive its blood supply by the testicular artery. It originates from the abdominal aorta, and elongates more than necessary to compensate for the migration of the testis into the scrotum. As a consequence, it becomes extensively coiled before birth [10]. The testicular artery appears convoluted just before its entry to the testis. This convoluted part is termed supratesticular artery (STA) [9]. The vascular resistance in this vessel is therefore high, leaving capillary pressure in the testis lower than other organs [11]. The oxygen tension in the testis is low. The high metabolic activity in the seminiferous tubules is apparently adapted to this environment of low oxygen and low vascular perfusion pressure, and under normal conditions, the local vasculature is able to supply the testis with sufficient amounts of nutrients and oxygen [11].

Doppler ultrasonography has become the method of choice to evaluate blood supply of various organs. It is one of the simplest and most precise techniques for estimating blood flow, as it combines data concerning the anatomy and dynamic flow parameters [12]. Doppler effect was first described in 1842 by the Austrian physicist Christian Johann Doppler. When ultrasound contacts static surfaces, without movement, the frequency of the reflected echoes is the same as that of the emitted waves. On the contrary, when the waves collide with moving structures such as blood cells, the frequency of the reflected waves is different from that of the emitted waves, producing the well-known Doppler effect. The change in frequency of the reflected wave with respect to the emitted wave can be positive or negative [13,14,15]. There are two types of Doppler, continuous and pulsed [14]:

- Continuous Doppler: This type does not discriminate depth of field. All the blood vessels in the area are captured, regardless of the range of ultrasound applied.
- Pulsed Doppler: With this system you can choose the depth of the field to study, specifically evaluating the vessels in that area.

There are three different ways of representing the Doppler signal as an image: Color Doppler, Spectral Doppler and Power Doppler [14]:

- Color Doppler (CD): Provides a general idea of the presence and direction of blood flow. It encodes the frequency changes in red and blue. If the frequency is positive, the image will be red, while if it is negative, it will be blue. In addition to color, the brightness of the image is taken into account; the higher the brightness, the greater the frequency amplitude [14,15,16].
- Spectral Doppler (SD): Frequency changes are plotted on a graph as a function of time [13]. Analyses the speed of blood flow in a vessel, illustrating its hemodynamic [14,15,16].
- Power Doppler (PwD): It is the newest method. Unlike the others, it does not measure the speed or direction of the flow, but the intensity. It measures the number of cells that pass through a glass in a unit of time. The images are represented in color mode (orange-brown) in B mode. PwD is used in areas where blood flow is very slow, such as ovarian follicles or corpora lutea [17,18].

The use of Doppler ultrasonography in animal reproduction research is more recent than in human research but not less significant. In animal reproduction, the significance and feasibility of the use of Doppler sonography was first used to study the vascularization of the female reproductive tract. Several studies have evidenced, through Doppler sonography, the relationship of blood flow and ovarian and uterine function throughout the estrous cycle and pregnancy [13].

The study of testicular hemodynamics is of great significance; in fact, one study that studied the blood flow in the testicular artery above the cord and on the surface of the testis in dogs showed that at both sides there is only about a 30% reduction in blood flow velocity between systole and diastole, compared with more than 90% in prostate artery [10]. This situation suggests that moderate disturbances in the blood supply to the organ could cause testicular malfunction [11].

Several researchers have studied the effect of reductions in testicular blood flow in spermatogenesis. Induced partial arteriosclerosis in the testicular artery of rams severely affected the seminiferous tubules, leading to an inflammatory reaction similar of that seen in infertile men [17]. In rats, it was observed that graded reductions of blood flow affects the early stages of spermatogenesis. When blood flow was reduced, there was a dose-related increase in the number of dying spermatogonia and early spermatocytes together with an increase in the number of PMN leukocytes accumulated in testicular blood vessels. Discrete reductions in flow may, therefore, have a large impact on sperm production [11].

In human medicine, the study of testicular vascularization by duplex Doppler ultrasonography is well established [18]. PwD ultrasonography is widely used to assess abnormalities of the male genital tract, and has proven to be a useful tool in the assessment of scrotal abnormalities, signs of testicular dysgenesis, inflammation of scrotal structures, testicular torsion and varicocele [19]. In veterinary medicine, the study of testicular vascularization was initially restricted to horses. Pozor and McDonnel (2004) concluded it is possible to characterize the testicular artery by ultrasonography and the importance of its measurement in two locations due to its convoluted course [20]. Following these results, more research was performed in other animal species, establishing physiological parameters of the testicular artery using CD and SD at different locations in stallions [20,21,22], jackass [23], dogs [21,24,25], felines [26], bull [27,28,29], rams [30,31,32,33,34], bucks [9,12] and camelids [35].

The testicles require a stable blood supply for its function and maturation [36]. CD and SD of the blood flow towards the testicular parenchyma could reflect the functional changes occurring in the different reproductive periods [12].

**Figure 1:**
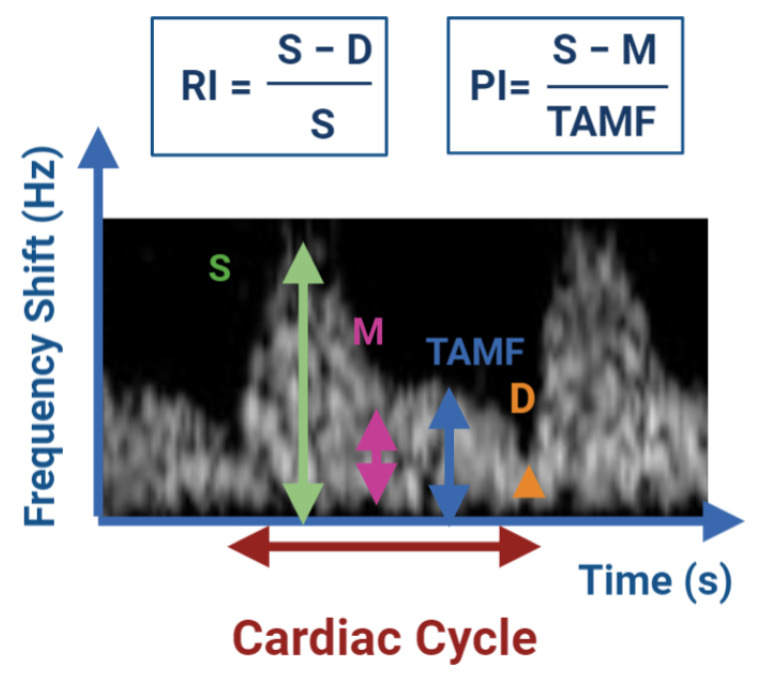
Semi-quantitative evaluation of blood flow by determination of the resistance to blood flow using the Doppler indices RI and PI. D: EDV; M: minimum frequency shift; S: PSV; TAMF: time averaged maximum frequency shift (TAMAX). Image adapted from Bollwein et al. [15].

The evaluation of the ovarian follicles and corpora lutea is most often not quantified by examining individual vessels, but by measuring the total area of colored pixels [15]. In the case of testicular vascularization, the internal spermatic artery and its branches are the major source of blood supply to the mammalian testis and epididymis [17]. In this case, the blood flow is evaluated in an individual vessel and it is typically evaluated semi-quantitatively using the Doppler indices [15]. These indices have been used to obtain information about blood flow and vascular impedance that cannot be obtained from velocity information alone. They depend on the measurements of the Peak Systolic Volume (PSV), End Diastolic Volume (EDV) and the mean velocity. The two widely used indexes are the Resistive Index (RI) and Pulsatility Index (PI) [36]. They are not a direct measure of blood flow, but rather describe the resistance to blood flow in vessels peripheral to the vessel being examined [15]. Figure 1 shows the typical waveform appearance of the spectral Doppler of one cardiac cycle in a buck and the parameters used for the semi-quantitative evaluation of the Doppler indices.

Decreased values for RI and PI indicate decreases in the blood flow resistance and in turn increase in the testicular perfusion and continuous supply of oxygen and nutrients to the testis [14]. For the measurement of testicular blood flow, the RI has been used to date in both animals and humans [36].

**Figure 2:**
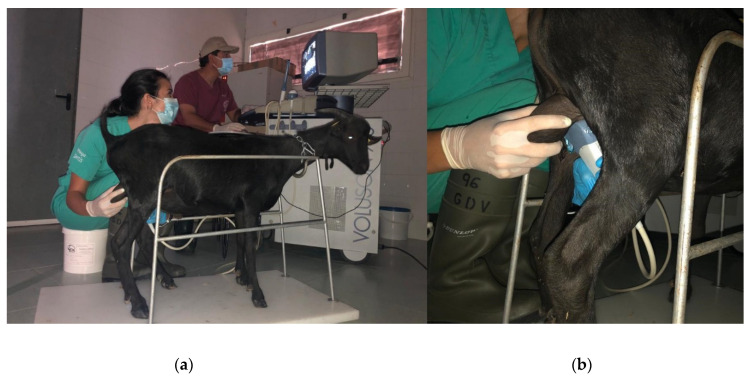
Correct Restraining (**a**) and ultrasound probe positioning (**b**) of a Murciano-Granadina buck for the Doppler ultrasound examination. Date: 3 July 2020.

When performing Doppler ultrasound examination, several aspects related to the immobilization of the animals, the placement of the probe and the correct identification of the testicular artery, must be taken into account to obtain valid results. A correct restraining of the animals is necessary to perform an adequate ultrasound exam since any movement may impair the correct caliper positioning during the exam [13,14,15]. In Figure 2*,* we can see an example of a correct restraining and probe positioning in a buck.

**Figure 3:**
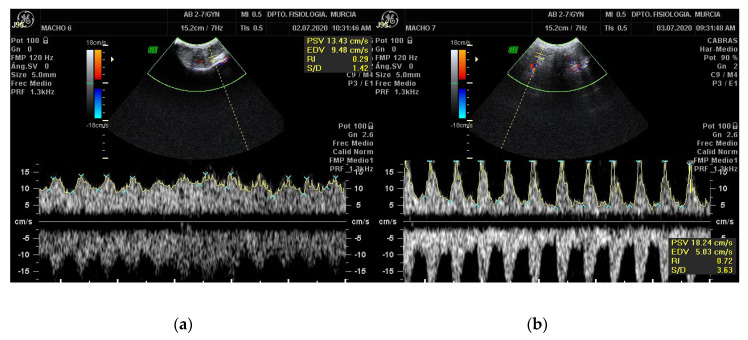
Pulsed-wave Doppler of the STA of a Murciano-Granadina breed buck. The pictures show the appearance of the pulsed-wave Doppler ultrasonography of the testicular vein (**a**) and of the testicular artery (**b**). Date: 3 July 2020.

In the Doppler velocimetry assessment of the testicular artery, firstly, two-dimensional color Doppler scans have to be initially performed to visualize the testicular artery in the spermatic cord at the desired location. Secondly, blood vessels and flow must be identified using CD. Red indicating blood flow towards the transducer and blue indicating blood flow away from the transducer both in left and right testis [18]. Veins surround the STA forming the vascular cone. Therefore, before the assessment of Doppler parameters of the artery, a clear differentiation between the artery and veins should be done. To differentiate them, SD should be performed, the artery will typically have a spectral waveform representing the arterial pulse in each cardiac cycle. In the vein, however, the flow has no pulse and it is almost constant, as shown in Figure 3. After the identification of the vascular structures, the largest longitudinal section of the testicular artery at different locations must be identified. Then, the Doppler caliper has to be placed on the vessel lumen, and the arterial blood flow will be graphically represented as a wave. A minimum of three waves must be chosen and measured [34,37,38] Furthermore, the Doppler shift is affected by the insonation angle between the Doppler beam and the flow direction [16,18,19]. The angle between the probe and the artery must be lower than 60° so that the velocity values are reliable. Ideally the insonation angle should be between 45° and 60° [14].

### 1.1. Relationship between Spermatogenesis and Doppler Ultrasonography Parameters

**Figure 4:**
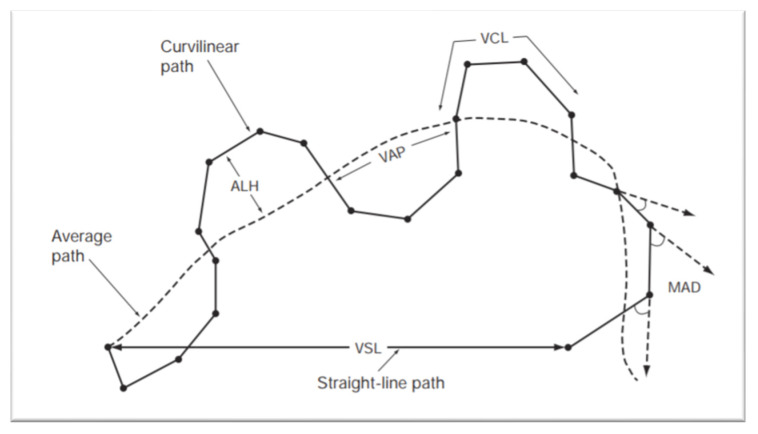
Standard terminology for variables measured by CASA system. Abbreviations include: Amplitude of lateral head displacement (ALH), average path velocity (VAP), curvilinear velocity (VCL), mean angular displacement (MAD), straight-line rectilinear velocity (VSL). World Health Organization Manual for the Evaluation of Human Semen [44].

The prediction of sires´ reproductive performance is of major economic importance in the animal breeding industry. However, semen analysis does not always correlate with field fertility outcomes [39]. Despite controversy regarding the clinical value of semen analysis, male fertility investigation still relies on a standardized analysis of the semen parameters [40]. The evaluation of semen is an essential part of the andrological screening and helps to identify clear-cut cases of infertility. Usually, the sperm analysis includes recording of the volume, appearance, sperm concentration, sperm morphology and motility [41]. As standard semen analysis is a rather subjective technique, tools for computer-assisted semen analysis (CASA) have been developed. By use of CASA several specific motility parameters describing the movements of spermatozoa in a more detailed manner can be obtained [42]. The various individual movements that can be evaluated by CASA are shown in Figure 4, and include: curvilinear velocity (VCL), straight-line rectilinear velocity (VSL), average path velocity (VAP), velocity index (SVI), amplitude of lateral head displacement (ALH), linearity of curvilinear path (LIN), wobble (WOB), straightness (STR), beat-cross frequency (BCF) and mean angular displacement (MAD) [43].

The clinical andrological examination of a sire aims not only to determine the normality of testicular and epididymal function, as well of that of the genital tract of the male but also to estimate its potential capacity as a breeder [41]. Several researchers have studied the predictive value of arterial impedance of the testicular artery in relation to sperm analysis [45].

In human medicine, Biagiotti et al. (2002) investigated the differences in EDV, PSV, RI, follicular stimulating hormone and bilateral testicular volume in different groups of fertile and infertile men. Only RI and PSV were significantly associated with the sperm production rate score [45]. Pinggera et al. (2008) suggested that an RI > 0.6 in humans might be related to a pathological sperm count in andrological patients [36]. In another study in varicocele patients, a negative association was found between progressive motility of spermatozoa and RI of the intraparenchymal arterial blood flow [46].

In veterinary medicine, the first attempts to study SD ultrasonography in the testicular artery were done in stallions. Pozor and McDonnell (2004) investigated the potential use of SD to characterize blood flow to the testis of stallions and to stablish reference values [20]. Bollwein et al. (2006) studied the relation between SD parameters and sperm quality. Blood flow was quantified using the PI and blood flow volume (BFV). No relationship was found between PI and sperm quantity or quality, although a connection was noticed between BFV and total sperm count per ejaculate [22]. Later on, Ortiz-Rodriguez et al. (2017) investigated the value of CD as a tool to evaluate testicular dysfunction in stallions. They found statistically significant differences in RI and PI values between fertile and sub fertile stallions, the latter group having higher RI and PI values. In contrast, fertile stallions displayed high EDV, TAMV, and BFV [21]. Similarly, in the jackass, RI and PI were strongly connected with CASA motility variable STR and sperm viability. Sperm concentration was inversely associated with PSV, EDV and TAMV. EDV was also inversely associated with CASA motility variables (VSL, LIN, STR and VAP) [23].

Several studies have characterized and established reference values of blood flow to the testicles in the dog [18,24,25,47,48]. Zelli et al. (2013) discovered a negative connection between PI and RI and the total progressive motility as well as a negative connection with the percentage of membrane intact sperms [47]. PSV was negatively associated with the number of live sperm [47]. This results contrast with those obtained by England et al. (2005) who failed to evidence a connection between RI and PI and the total sperm output or the percentage of live normal sperm [24]. In 2019, Trautwein et al. found a relationship between PSV with the velocity index, VCL and VAP [49]. In 2020, Lemos et al. found a relationship between PSV and EDV with sperm concentration as well as an inverse association between RI and PI at the STA with sperm membrane integrity. Additionally, a connection between EDV at the STA level with sperm concentration and an inverse association with sperm oxidative DNA damage were evidenced [25].

In the case of ruminants, the connection between CD ultrasonography parameters and future sperm quality was firstly assessed in the bull. A relationship was found between the RI of the marginal testicular artery (MTA) and total sperm in the ejaculate, immature sperm, teratoid sperms or “dag effect” spermatozoa [28]. In rams, no connection was found between motility and hemodynamic characteristics, but an association was found between the percentage of total sperm defects and RI and PI [33]. In bucks, Samir et al. (2020) demonstrated that the administration of melatonin decreased the RI and PI of testicular arteries, which was associated with significant increases in acrosome integrity, live/dead ratio, motility, and normal sperm morphology [9].

In camelids, differences in Doppler velocimetric parameters between fertile and infertile specimens were found. Fertile males yielded higher PSV and EDV values within the STAs. However, no significant differences between the RI of fertile and infertile males were found [35]. Previously reviewed results are presented in Table 1.

There is currently no proposed explanation for the positive relationship between RI and sperm count. Testicular arteries are target organs for androgens, and, in infertile men, testicular arteries have a narrow lumen due to enlarged endothelial cells, a thickened subendothelial layer and abundant adventitia rich in connective tissue fibers and ground substance [36]. The implication is that anatomical patterns of testicular arteries are related to spermatogenesis and that SD ultrasonography traces from the testicular artery can be considered as markers of spermatogenesis [36]. Studies where no association between SD ultrasonography parameters and seminal parameters was found justify this result, based on the hypothesis that the relationship of the testicular artery blood flow is more likely to be related to current rather than to future semen quality, due to the fact that endothelial thickening and changes in blood flow normally occur secondarily to testicular disease, while primary restriction of testicular artery diameter results in rapid testicular changes [24].

Another possible explanation for the differences found between studies performed in the same species can be due to the different vessels that were analyzed. Differences of Doppler velocimetric parameters have been assessed depending on the segment of the testicular artery which was evaluated [23,31,48]. Values also show discrepancies depending on the method used for semen recollection, for example in rams electroejaculation normally results in larger volumes and lower concentration [33].

### 1.2. Influence of the Location of the Assessment on Doppler Indices and the Spectral Doppler Waveform

In several animal species, up to five measurements at different locations of the testicular artery have been done [48,50]. The results of EDV, RI and PI testicular values are influenced by the location of the assessment. The locations that have been studied include proximal STA, distal STA, MTA and intratesticular [18]. In humans, the RI for capsular and intratesticular arteries were smaller than in the STA, while in stallions, values of mean RI in the marginal aspect of the artery were only slightly lower than in the convoluted aspect [23,51]. In dogs, Doppler velocimetry parameters show differences between the spermatic cord, MTA and intratesticular regions, with decreasing velocities as it approaches to testicular parenchyma [48,50]. PSV and EDV show a decreasing trend from STA region to the intra-testicular region, this changes resulted in a decreased RI and PI within the MTA and intratesticular artery [48].

**Figure 5:**
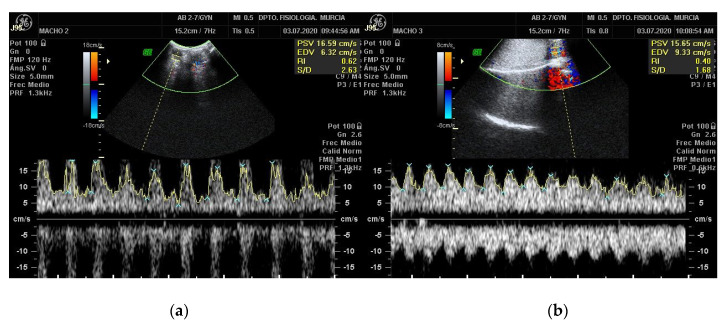
Ultrasonographic scan of the caprine testis using spectral pulsed-wave Doppler ultrasonography showing monophasic non-resistive wave form in the right STA (**a**) and in the MTA (**b**). Date: 3 July 2020.

Figure 5 shows a CD image of the STA and MTA. On pulsed-wave Doppler imaging, the testicular artery shows mostly a monophasic and non-resistive waveform pattern in dogs [47], rams [30] and goats [34]. However, SD imaging of the testicular artery in stallions showed a resistive biphasic waveform [20]. These differences are believed to derive from the position of the testes on its long axis, being oriented horizontally in horses, vertically in ruminants and cranio-ventrally in dogs [30].

Regional differences in wave morphology have been described. In dogs the waveform evolves from a semi-parabolic with high resistivity to intermediate in the cranial portions of the testicular artery to parabolic with low resistivity in the caudal and intratesticular portion. In stallions, an individual variation of waveform at different locations was evidenced. In general, the waveforms at the convoluted aspect of the TA were mostly resistive biphasic and evolved to non-resistive at monophasic at the MA level [20]. These findings can be explained by the fact that the testicular artery originates directly from the aorta, a vessel with high resistivity to blood flow. This resistivity decreases due to its prolongation and tortuosity with reduced thickness of the vascular endothelium as it approaches the testis. This directly influences the RI and PI [50].

### 1.3. Influence of Breed and Size on Doppler Indices

In many species of livestock, we can find breed differences with respect to the age of puberty, ejaculate volumes, sperm concentrations, and total sperm per ejaculate [52]. Little research has been done evaluating the effect of the breed on blood flow dynamic. It is hypothesized that animals with different genotypes could exhibit different physiological parameters of testicular hemodynamic. In fact, Junior et al. (2018) compared Doppler values of STA blood flow pattern in different breeds of bulls, the study found differences of the mean velocity, PI and RI between Aberdeen Angus, Bradford, Brangus, Hereford and Nelore bulls [27]. In another study performed in Nelore and Caracu bulls, significant differences between RI and PI where also found, meaning biometric ultrasonography characteristics of the testicular artery are affected by the genetic group [29]. The size of the animal also affects Doppler velocimetric parameters. Souza et al. (2014) evaluated Doppler velocimetry parameters in dogs of different sizes. Larger dogs showed higher velocities (EDV) at spermatic cord, while small dogs presented higher EDV at the level of the MTA. This difference might be explained by the length of the TA, which may vary according to weight [53]. When examining an animal, its breed and size should be taken into consideration when looking at the results.

### 1.4. Influence of Season in Testicular Blood Flow Dynamics

A large majority of farm animal express a more or less marked seasonal reproductive stationarity. The degree of reproductive seasonality expressed by an animal can vary markedly in intensity and timing. These variations depend on several factors including environmental factors, species, gender, genotype, reproductive status and amount of body energetic stores among other factors. As a consequence, most species show seasonal variations in their ovulation frequency, spermatogenic activity, gamete quality and also sexual behavior [54,55]. Some of the species which show a marked seasonality in breeding are small ruminants and equines [2,23].

In seasonal breeders, the volume of the ejaculate is high in the breeding season and decreases in the non-breeding season. Sperm concentration follows the opposite trend [54]. In 1999, Karagiannidis et al. described differences in seasonal semen quality in three dairy goat breeds born and raised in Mediterranean countries. There was a clear effect of photoperiod on their semen production [56]. These changes have also been noticed in ultrasonography. In goats, the testicular volume increased during the summer months reaching its highest values in September [12]. Similarly, in rams the mean testicular pixel intensity reached its lowest values during the winter and increased gradually reaching its highest values during summer [57].

Few studies have been conducted regarding annual variations in the SD ultrasonography parameters in mammals. Variations in SD ultrasonography parameters along the year were first described in stallions, in which Doppler parameters related with increased perfusion (PSV, EDV and TAMAX) presenting higher values during breeding season [58].

In caprine, to our knowledge, only two studies have been performed that describe annual variation in SD ultrasonography parameters. The first one was done in Sarda bucks by Strina et al. (2016). A decrease in the mean values of the RI of the testicular artery in the summer months was described, while the highest value of RI was detected in November [12]. The second experiment was performed in Shiba goats by Samir et al. (2018), described as non-seasonal breeders. Variations in the RI and PI were found, in opposition to the previous results the lowest values of PI and RI were found during winter and autumn [2].

Changes in the testicular blood flow among the seasons are attributable to two possible mechanisms, either due to variations in ambient temperature between the seasons or due to photoperiod, which is considered the principal factor influencing seasonality of reproduction in small ruminants [2]. In the summer season, increases in ambient temperature may result in concurrent increases in testicular temperature and, in turn, affect the testicular blood flow. In bulls submitted to short term testicular warming under anesthesia, the total blood flow was measured using a ultrasonic flow prove around the testicular artery and the testicular vein. Increases in testicular temperature and blood flow were related in Angus and Nelore bulls [59]. In rams, the blood flow to the testis was calculated using the technique of dilution of sodium p.aminohippurate. The blood blow increased spontaneously a 26% when their testis were exposed to direct heat [60]. In the study of Strina et al. (2016) a coincidental variation in testicular flow due to changes in ambient temperature may be the possible explanation to their results [12].

### 1.5. Effect of Laterality in Testicular Blood Flow Dynamic

Differences in testicular volumes on the left and right tests have been recorded in several animal species. In dogs, the PSV of the left spermatic cord was significantly higher than the right side [18]. Hedia et al. (2020) found that Doppler measures of RI as well as PI were slightly higher in the right testicular artery rather than the left testicular artery in rams [30]. In horses, no significant differences in RI, PI EDV and PSV were found between the left and right artery [20]. This difference might be explained because the left testicular artery arches over the renal vein, compressing the vessel and increasing velocity of blood flow in this area. The difference can also be explained due to the differences found between the volume of the left and right testis; a higher volume would require more blood supply [18].

### 1.6. Limitations of the Use of Doppler in BSE

The main limitation of this study is the workability. Differently from human medicine, the adoption of Doppler imaging technology in large animal practice is still limited. The two main factors that have contributed to this low use of Doppler technology at the farms is the availability and cost of portable devices and the relative lack of knowledge of practitioners [13]. Other possible limitations of this study include the fact that most of the objective measurements of blood flow performed by Doppler ultrasonography including the calculation of flow velocity and indices are usually performed in large straight arteries [13]. One example in human medicine is the common carotid artery [16]. The evaluation of the STA is especially challenging due to its coiled section before the entrance to the testis. As the STA is intertwined with the testicular vein it is sometimes difficult to assess differences between testicular artery and vein in the Doppler analysis [2]. Any movement of the animal may also impair the correct caliper positioning during the exam. Additionally, the Doppler shift is affected by the insonation angle between the Doppler beam and the flow direction [13,14,15]. Blood flow velocities measured as the PSV or EDV represent an angle dependent technique, so careful interpretation must be performed. Using the RI to assess blood blow represents the best option as it is independent of the angle [36].

## 2. Conclusions

Improving the reproductive performance of production animals continues to be one of the leading objectives in animal production research. SD has been used in humans and several animal species to objectively evaluate blood flow to the testes. In humans, studs, bulls, stallions, jackass, buck, rams and camelids, several SD velocimetric parameters including EDV, PSV, RI and PI have been associated with various parameters related to a better seminal quality. These results suggest SD ultrasound parameters could be proposed as objective parameters to evaluate testicular function and should be taken into consideration when performing the breeding soundness evaluation. Because of the variability found in these parameters depending on the location of the measurement, season, breed and laterality. Further research is needed to determine SD physiological parameters according to the species and breed, establishing reference values for the species within specific sizes.

## Figures and Tables

**Table 1:** Present research results proving the relation between arterial Doppler velocimetric indexes and seminal quality in domestic animal species.

Species	Relation between CD Ultrasonography Parameters and Seminal Quality
Bovine	RI at the MA and IT associated with total sperm per ejaculate, inmmature sperm, teratoid sperm and dag effect sperm. Gloria et al. [28]
Ovine	RI and PI at the STA were associated with the total sperm deffects. Batissaco et al. [33]
Equine	Relationship between BFV at the STA and total sperm per ejaculate. Bollwein et al. [22]
	Infertile stallions exhibited higher values of RI and PI at the MTA level. Ortiz-Rodriguez et al. [21]
Donkey	RI and PI at the capsular artery were associated CASA motility variables and sperm viability. Sperm concentration was inversely associated with PSV, EDV and TAMV Gacem et al. [23]
Canine	PSV had a inversed association with vitality. Negative connection between RI and PI with the percentage of intact sperm membranes. Zelli et al. [47]
	No association between RI and PI of the TA and total sperm output or vitality. England et al. [24]
	Positive association between PSV at the proximal STA with SVI, VCL and VAP. Trautwein et al. [50]
	PSV was associated with sperm concentration. RI and PI of the STA were inversely associated with sperm membrane integrity. EDV at the STA was associated with sperm concentration and inversely associated with sperm oxidative DNA damage. Lemos et al. [25]
Camelid	Fertile males exhibited higher values of PSV and EDV at the STA level and higher values of PSV at the MTA level. Kuzler et al. [35]

## Data Availability

No new data were created or analyzed in this study. Data sharing is not applicable to this article.
